# Effectiveness of one-to-one volunteer support for patients with psychosis: protocol of a randomised controlled trial

**DOI:** 10.1136/bmjopen-2016-011582

**Published:** 2016-08-03

**Authors:** Stefan Priebe, Hana Pavlickova, Sandra Eldridge, Eoin Golden, Paul McCrone, Nick Ockenden, Nancy Pistrang, Michael King

**Affiliations:** 1 Unit for Social and Community Psychiatry (WHO Collaborating Centre for Mental Health Services Development), Queen Mary University of London, London, UK; 2 Pragmatic Clinical Trials Unit, Queen Mary University of London, London, UK; 3 Department of Clinical, Educational and Health Psychology, University College London, London, UK; 4 Institute of Psychiatry, Kings College London, London, UK; 5 Institute for Volunteering Research, National Council for Voluntary Organisations (NCVO), London, UK; 6 Division of Psychiatry, University College London, London, UK

**Keywords:** befriending, social inclusion, psychosis, community care, volunteer

## Abstract

**Introduction:**

Social isolation is common in patients with psychosis and associated with a number of negative outcomes. Programmes in which volunteers provide one-to-one support—often referred to as befriending—have been reputed to achieve favourable outcomes. However, trial-based evidence for their effectiveness is limited.

**Methods and analysis:**

This is a randomised controlled trial comparing the effects of one-to-one volunteer support with an active control condition for patients with psychosis over a 1-year period. Patients in the intervention group will receive the support of a volunteer for 1 year, who will meet them weekly and engage them in social and recreational activities. Patients in the control group will not receive support from a volunteer. In both groups, patients will be given a booklet detailing locally available social activities and otherwise receive treatment as usual. Patients, volunteers, clinicians and researchers involved in the delivery of the intervention will not be blinded to group assignment, while researchers carrying out data collection will be blinded. Data collection will be conducted at baseline, at 6 and 12 months. The primary outcome is the amount of time spent engaging in social activities per day. Secondary outcomes include symptoms, quality of life, self-esteem and costs of care. Attitudes of volunteers towards mentally ill people will be assessed. Finally, in-depth interviews will be conducted with patients and volunteers.

**Ethics and dissemination:**

The study has been approved by the National Research Ethics Service (NRES) Committee London—Camden & Kings Cross (reference 15/LO/0674). The findings of the trial will be published in open access peer-reviewed journals and in the National Institute for Health Research (NIHR) journals library, and presented at scientific conferences. In addition, findings will be summarised for a lay audience and circulated to all relevant National Health Service (NHS) and voluntary organisations.

**Trial registration number:**

ISRCTN14021839; Pre-results*.*

**Strengths and limitations of this study**

- The randomised controlled trial is rigorously designed to address the gap in evidence regarding the effectiveness and cost-effectiveness of one-to-one volunteer support for patients with psychosis.
- Experiences with the intervention of patients and volunteers will be examined.
- The findings may inform the practice of volunteering programmes for community patients with psychosis within different types of organisations.
- A number of outcomes are assessed. However, there may be different perspectives on what the most relevant outcomes of volunteer support are.
- The nature of the study poses a high risk of unblinding of research interviewers.

## Background

Social isolation of patients with psychotic disorders remains one of the main challenges in community mental healthcare. It has been linked to poorer clinical outcomes with respect to mental and physical health, life expectancy, engagement with treatment, and quality of life.^1^ ^2^

Over the past decades, both the National Health Service (NHS) and the independent sector in the UK have used volunteer resources to support mental health patients in a wide range of areas, including so-called befriending. Although the format of befriending varies across organisations, it typically involves volunteers spending recreational time with the patient over a period of time, so as to provide them with an opportunity to socialise. This is different to so-called ‘peer support’ where patients provide support to one another, and the effects operating within such interactions might differ from those employed in befriending. Volunteers are a valuable asset to mental healthcare, uniquely complementing service provision where mental health professionals may fall short. Both clinical and social gains of volunteer support have been reported. Patients receiving befriending have reported short-term reduction of symptoms,^3^ ^4^ improvements in confidence and self-esteem^5^ ^6^ and increased energy and interest in going out.^7^ However, trial-based evidence on the effectiveness of these one-to-one support schemes is limited.

Against this background, we designed a randomised controlled trial (RCT) aiming to test the effectiveness and cost-effectiveness of a programme of one-to-one volunteer support for patients with psychosis. The programme is referred to as a companion scheme to avoid the term befriending misleading patients into expectations of a true friendship where some boundaries to the relationship are imposed by the scheme.^8^ The scheme is based on best practice principles from befriending organisations gathered in a study in the UK (J Silvester, S Priebe, N Giatras, *et al*.s Reflecting on rhetoric and practice in the use of human resource management with volunteers. Human Relations in preparation)^8^ and will be implemented over a 1-year period. The intervention will be compared with an active control condition. It is hypothesised that volunteer input will result in patients' increased social and recreational activities, beyond those arising directly from the time spent with the volunteer, and potentially also be beneficial with respect to social contacts, symptoms, quality of life and self-esteem.

## Methods

### Study setting and design

**Figure1:**
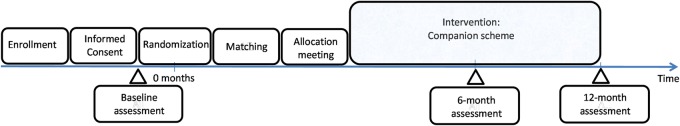
Time schedule.

This is a parallel group exploratory RCT with 1:1 allocation ratio testing the effectiveness of volunteer support, specifically for patients with psychotic disorders with limited engagement in social activities. The trial compares the intervention, regular one-to-one support from a volunteer, to an active control condition in which there is no support from a volunteer. Data are collected at baseline, 6 and 12 months (figure 1). Researchers conducting outcome assessments will be blind to the allocation of patients. All the statistical analyses will be conducted blind to the participant allocation. The trial is conducted within community mental health setting in the UK.

### Participants

#### Inclusion and exclusion criteria for patients

Inclusion criteria for patients are a diagnosis of schizophrenia or a related disorder (International Classification of Diseases (ICD)-10: F20-29); engaging in an average of <60 min per day of social activities, as assessed by the Time Use Survey (TUS);^9^ willingness to receive regular one-to-one input from a volunteer over a 1-year period; working age (18–65 years); capacity to provide written informed consent; sufficient spoken English and being in care of a community mental health team for a minimum of 1 month.

Patients are excluded if they have a severe learning difficulty, or physical disability that would require specific skills of a volunteer; have received befriending or peer support services in the past 2 years; or have a history of physical violence or abuse posing a risk to the volunteer, as assessed by the referring clinician.

#### Inclusion and exclusion criteria for volunteers

Inclusion criteria for volunteers are being at least 18 years of age; willingness to provide regular one-to-one support to a patient with a psychotic disorder over a 1-year period; sufficient command of English and criminal records showing no major convictions (eg, unspent convictions of fraud or theft; convictions of violent assault or sexual offences).

Exclusion criteria are the use of secondary mental health services as a patient currently or in the last year; having a professional role in a secondary mental health service; severe physical disability interfering with social activities; and insufficient understanding of the responsibilities and characteristics of a volunteer despite having undergone volunteer training.

### Recruitment

#### Patients

Eligible patients are identified by blinded researchers screening caseloads with clinicians in mental health teams in the community in East London, which began in July 2015. They are then approached by their clinician and asked whether they are willing to be contacted by a researcher to learn more about potentially becoming involved in a companion scheme. If they agree, a meeting is arranged at which adequately trained researchers in obtaining informed consent provides the patient with a detailed explanation of the study and a participant information sheet. If the patient agrees to participate, they are asked to sign a consent form. Their demographic information is collected and they are screened using the TUS. If the patient is found to engage in more than 60 min of social activities per day on average over the previous 4 days, they are informed that they are not eligible for the study and given £5. If the patient engages in fewer than 60 min of social activities per day, a full baseline assessment is conducted, and they are given £15 for their participation in the research interview. They receive the same amount for any subsequent follow-up interview.

Adequate enrolment will be achieved by approaching a number of community mental health teams across East London (including the London Boroughs of Hackney, Newham and Tower Hamlets). Enrolment progress will be regularly monitored, barriers identified and if needed advised sought with the Trial Steering Group.

#### Volunteers

Volunteers are recruited through advertisement of the companion scheme on job websites, volunteering websites and the volunteering section of the East London NHS Foundation Trust website; advertisement in local newspapers; information stalls at volunteering fairs and poster displays in local community centres in East London.

Interested individuals are invited to phone or email the volunteer coordinator, who provides information about the companion scheme and its requirements, including the 12-month commitment and eligibility criteria. They are provided with a participant information sheet explaining the research linked to the companion scheme and their role as research participants, and asked to complete an application form. After completing the form, they are invited for a meeting with the volunteer coordinator. At the start of the meeting, informed consent to participate in the research is taken and they complete the baseline assessment. They are informed that this assessment is for research purposes and that their answers will not have any bearing on whether or not they are accepted to participate in the scheme. The assessment collects demographic information and assesses attitudes to mental illness, and the person's self-esteem. They are given £10 for the completion of the baseline measures and receive the same amount for any subsequent follow-up interview.

On completion of the baseline, the volunteer undergoes an informal, standardised interview carried out by the volunteer coordinator to assess their suitability for the scheme. This interview explores their motivation, empathy, relationship building, organisation and communication skills, identified as key qualities in volunteers in an earlier research component of the wider research programme (J Silvester. ‘In preparation’). After this interview, they provide the necessary information for a Disclosure and Barring Service (DBS) to be undertaken. Provided that there are no issues with the DBS that would deem it unsuitable for the volunteer to work with vulnerable adults, and on receipt of two references in support of their application, they are accepted to the scheme.

### Planned interventions

#### Experimental intervention

This consists of individual patients with psychosis being enrolled in a companion scheme, whereby they are matched with a volunteer companion, who will provide one-to-one support on a weekly basis over a 1-year period. This is in addition to their treatment as usual. The main aim of the scheme is to gradually develop a trusting relationship and motivate patient to engage in social activities within local community. This will involve chatting over a cup of tea at the beginning and develop into more active social engagement such as attending workshops and events available in the local area according to patient's interests. The companion scheme is a complex intervention comprising the recruitment of suitable volunteers, their training and supervision (described below), and the regular contact between them and the patients. The format of the scheme has been informed by interviews with participants in existing befriending schemes (befrienders, befriendees and coordinators of schemes) as part of the wider research programme.

For each patient–volunteer pair, the volunteer coordinator attempts to create a match based on common interests and individual preferences, as well as practical considerations such as distance and mutual availability. A meeting is arranged to introduce the pair at the patient's home, unless the patient wishes for the meeting to take place elsewhere. This is treated as a ‘test meeting’, after which the volunteer coordinator confirms with both parties separately that they are willing to proceed with the match. The volunteer coordinator facilitates the meeting by explaining the purpose of the meetings, boundaries and ground rules and establishing common interests among the two. Both parties are provided with an activity booklet detailing free or inexpensive facilities and activities available locally (eg, parks, community centres, libraries, sports, leisure centres, galleries and museums) and are encouraged to pursue these during their contact. It is reiterated that the pair should meet weekly for at least 1 hour, and that these meetings will continue for 1 year, after which they can decide whether or not they wish to continue to meet, without being overseen by the companion scheme.

The pair subsequently meet on a one-to-one basis, without being accompanied. The volunteer coordinator provides supervision by contacting both parties within the first month and intermittently thereafter to monitor progress and identify any problems. If either party wishes to discontinue the match, the coordinator hears from both parties and attempts to resolve any issues where possible, and arranges to facilitate a further meeting between the two. If one or both parties still wish to discontinue the match, attempts are made to match both parties with another person, where practically feasible. If either party wishes to discontinue their participation in the scheme, the volunteer coordinator attempts to establish whether anything can be done to aid their continued participation, prior to formally withdrawing them from the scheme.

Every 3 months, all patients and volunteers are invited for a social meet-up (eg, brunch at a local café, a walk and picnic in a nearby park) with the aim of facilitating additional interactions between patients and volunteers. Patient–volunteer pairs are actively encouraged to meet with other pairs to facilitate wider networks and reduce the focus on an exclusively one-to-one relationship.

##### Volunteer training and supervision

Volunteers deemed suitable for the scheme (see the Volunteers of Recruitment section above) attend a mandatory training session across 2 days. The training provides a comprehensive understanding of the purpose of the scheme, and of the role of the volunteer. The importance of encouraging the patient to participate in local activities outside the home, such as those listed in the activity booklets, is emphasised throughout. Further aspects of training include understanding schizophrenia and related disorders, a questions and answers with a psychiatrist, a hearing voices simulation, a talk from a service user with lived experience of schizophrenia, maintaining confidentiality, procedures for safeguarding vulnerable people and seeking help in an emergency, communication and listening skills, and managing boundaries with the patient. In addition, practical matters such as claiming travel expenses and points of contact are addressed. On completion of training, volunteers are provided with a volunteer handbook covering all aspects of their participation in the scheme. Before meeting the patient with whom they will be matched, each volunteer is given a mobile phone and SIM card for communicating with the patient, paid for by the scheme. They are encouraged to contact the volunteer coordinator at any time. In the event of the volunteer reporting challenges in the relationship that cannot be resolved by the volunteer coordinator, they are offered a meeting with a consultant psychiatrist to discuss their concerns.

#### Active control condition

Patients in the control condition do not receive the support of a volunteer companion, and continue with treatment as usual. In addition, a meeting is scheduled with a member of the research team shortly after allocation to the control, during which the patient is given an activity booklet detailing free or inexpensive facilities and activities available in their locality (as given to patients and volunteers in the experimental condition). The main aim of the booklet is to inform patients of venues for active social engagement (eg, workshops offered in community centres and libraries, classes at sports clubs and leisure centres); however, activities with lower levels of social interactions are also included (eg, parks, galleries and museums). During the meeting, patient and researcher discuss the patient's interests and the researcher encourages the patient to attend suitable activities detailed in the booklet. This is intended to control for the effect of patients in the experimental group receiving more information about locally available activities via the volunteer.

### Outcomes

The following outcome criteria are measured at all time points (ie, baseline, 6 and 12 months), unless stated differently.

#### Primary outcome

- The primary outcome is the amount of time in minutes per day that is spent engaging in social activities, on average, over the previous 4 days. It is assessed using a shortened version of the TUS, developed by the Office for National Statistics (ONS) to examine how the general population in the UK spend their time. The measure has been previously used with patients with schizophrenia.^10^ The present version excludes any items related to social media interaction. A period of 4 days allows sufficient time for increased social activity to be detected, with reduced risk of recall bias.

#### Secondary outcomes

- The number of social contacts over the previous 4 days is assessed using the 16-item patient-rated Social Contacts Assessment (SCA; D Giacco, C Palumbo, N Strappelli, *et al*. Objective social isolation and loneliness in patients with psychotic and mood disorders. *Compr Psychiatry* under review).
- Subjective quality of life is assessed using the Manchester Short Assessment of Quality of Life (MANSA).^11^
- The objective social situation is assessed using the Objective Social Outcomes Index (SIX).^12^
- Both patient and volunteer self-esteem are assessed using the 20-item Self-Esteem Rating Scale—Short Form (SERS-SF).^13^
- The therapeutic relationship will be assessed using the Scale to Assess Therapeutic Relationships in Community Mental Health Care (STAR).^14^ Only patients in the intervention arm and their volunteers will complete this, 6 and 12 months after beginning the intervention. Patients will complete the patient version of the scale (STAR-P) while volunteers will complete an adapted version (STAR-V) of the clinician scale (STAR-C).
- Attitudes towards mentally unwell people will be assessed in volunteers using the Social Distance Questionnaire;^15^ and the Community Attitudes toward the Mentally Ill scale (CAMI).^16^

#### Clinical symptoms

- Patients' positive and negative symptomatology is assessed on the Positive and Negative Syndrome Scale (PANSS),^17^ a 30-item observer-rated measure, and the Clinical Assessment Interview for Negative Symptoms (CAINS),^18^ a 13-item observer-rated measure of negative symptoms.
- Patients' level of depression is measured using the Beck Depression Inventory (BDI).^19^

*Costs*: Service use and costs are assessed using the Client Service Receipt Inventory (CSRI).^20^ Quality of life-adjusted years (QALYs) are calculated based on the EQ-5D-5L.^21^

In addition, demographic information is collected at baseline. The number of meetings between each volunteer–patient pair and the amount of time spent together during each meeting is documented throughout the study.

### Qualitative data

At the end of the intervention, 20 patients in the intervention group and 20 volunteers will be asked to complete semistructured, in-depth interviews about their experiences of the scheme. We will employ purposive sampling to capture patients who completed the intervention as well as those who showed irregular patterns in meeting with volunteer. The interviews will be audio-recorded, transcribed and analysed using the thematic analysis framework proposed by Braun and Clarke.^22^ Participants will be paid £15 for their participation in the interviews.

### Assessment procedures

All researchers involved in data collection both prior and after intervention allocation are blinded for the duration of the trial, while it will not be possible to patients, volunteers, clinicians and researchers involved in the intervention delivery to be blinded to the allocation The only exception to this is data collection for the STAR-P and STAR-V, as these assess the relationship between the patient and the volunteer, which applies to the intervention group only. These assessments are carried out via phone by unblinded researchers involved in the delivery of the intervention.

There is a considerable risk of researchers becoming unblinded during follow-up assessments due to patients in the experimental group mentioning activities with the volunteer. To limit this risk, prior to each interview, patients receive instructions to avoid revealing their allocation. In addition, at the end of the interviews, researchers record their guesses as to whether patients are in the intervention or the control group.

There is also a risk that some measures are inflated in the intervention group by the intervention itself, that is, increased social activity as a direct result of meetings with the volunteer. To address this, unblinded researchers liaise with volunteers to ensure that no meeting takes place between the patient and the volunteer in the week prior to the follow-up assessment.

Guidance on assessment where participants provide ambiguous answers (eg, patients reporting having spent between 1 and 2 hours engaging in activities) has been developed and is adhered to by all researchers. Inter-rater reliability across researchers involved in data collection is established for PANSS and CAINS prior to first assessment.

All quantitative data are entered into Access databases, and will be retrieved for data cleaning before statistical analyses. Any personal information stored in locked cabinets on NHS premises if in paper version, and encrypted if in electronic version.

## Randomisation

Following baseline assessments, eligible patients are randomised either to the intervention condition or the active control condition, using a 1:1 block design algorithm via the online randomisation site of the registered Pragmatic Clinical Trials Unit (PCTU) at Queen Mary University of London. A one-off meeting is then arranged by an unblinded researcher to inform a patient of his or her allocation.

## Proposed sample size

Previous unpublished work within the wider research programme surveying 113 patients with psychosis in secondary care in East London has shown that patients tend to spend 327 min per week on average engaging in social activities (SD=480), or ∼45 min per day. Doubling this to 90 min per day would be equivalent to an effect size of 0.6 (Cohen's d). To detect such an effect on a 5% significance level with 80% power, data for 84 patients (2×42) are required. Assuming a 20% drop out rate between baseline and 1-year follow-up (as observed in similar trials with similar patient populations),^23^ a total sample size of 106 is needed.

## Data monitoring

All data are stored in accordance with the Data Protection Act 1998 and accessible only to members of the research team. Patient-identifiable data are anonymised and password-protected. The trial has no formal data monitoring; however, the Trial Steering Committee will provide input into data monitoring. No interim analyses will be carried out. No risk to participants is expected; however, any adverse events occurring during the study period will be recorded.

## Statistical analysis

### Quantitative assessments

All statistical analyses will be described in a statistical analysis plan, which will be finalised and agreed prior to any analysis or unblinding of the data. Outcomes will be compared between the intervention and control groups using linear regression models, adjusting for the baseline score of the given outcome.

All analyses will be conducted under the intention-to-treat principle, and significance testing will be at the 5% level (two-sided). Results will be presented in line with the recommendations given in the CONSORT statement extension for the reporting of RCTs.

#### Sensitivity analysis

Any outlying observations will be checked for data accuracy and may be excluded in a sensitivity analysis. Individuals may drop out of the intervention or be lost to follow-up. In these cases, assumptions must be made about their outcome data. One may argue that patients who drop out are more likely to do so if they do not perceive benefit of the intervention, and most likely would have ended with a less favourable outcome had they stayed in treatment. Yet, this is not necessarily so for patients with severe symptoms of schizophrenia. They might also tend to drop out when they perceive improvement and no further need for the treatment (in this trial, meetings with a volunteer), in which case drop outs would have ended with more favourable outcomes. Sensitivity analyses for both scenarios will be explored. Depending on data available, we will also conduct a per protocol analysis.

The final data set will be accessible to the principal investigator and the trial statisticians.

### Economic evaluation

The cost of recruiting, training and supporting volunteers will be estimated, and added to costs derived from the CSRI. This measure will record service use at baseline and each follow-up, and will combine this information with standard unit costs. Total costs will be compared at each follow-up point, controlling for baseline costs. A bootstrapped regression model will be used for this, given that the cost data are likely to be positively skewed.

Cost-effectiveness will be assessed by combining the follow-up cost data with the primary outcome measure and also QALY. The latter will be calculated using EQ-5D scores and standard weights attached to these. Incremental costs and outcomes will be used to define cost-effectiveness ratios (in the absence of dominance). Uncertainty around the cost-outcome combinations will be explored using cost-effectiveness planes, and interpretation will be aided using cost-effectiveness acceptability curves.

## Ethics approval and dissemination

Any changes to the protocol will be communicated to and approved by the Ethics Committee and the sponsor, and patients as appropriate. The findings of the trial will be published in open access peer-reviewed journals and in the National Institute for Health Research (NIHR) journals library, and presented at scientific conferences. In addition, findings will be summarised for a lay audience and circulated to all relevant NHS and voluntary organisations.

## Discussion

The present trial aims to provide evidence of the effectiveness and cost-effectiveness of one-to-one volunteer support for patients with psychosis. The trial is rigorously designed, including a well-defined and closely monitored intervention with systematic training and supervision of volunteers, an active control, and blinded outcome assessment.

Implementing the trial design has a number of challenges. Patients with psychotic disorders often have high levels of social withdrawal, but may not necessarily express a feeling of loneliness. Thus, their motivation to engage in social activities may be low, and they might either not be willing to establish a relationship with a volunteer or struggle to continue with regular meetings over a 1-year period. Matching of individual patients and volunteers is rather speculative, and there is no published research evidence available to guide this process. In addition, influencing the behaviour of volunteers with a view to improving patient outcomes may be more difficult to achieve than in trials targeting clinicians' actions.

There are also various challenges to the evaluation methodology. The primary outcome of assessing time spent on social activities might not capture the real extent of social isolation. The intervention may increase social activities, but ensuring that this increase is not inflated by the direct contacts with the volunteer may be difficult. Equally, patients' opportunity to socialise further may be restricted by the time spent with the volunteer. Measuring time use over the previous 4 days rather than the previous week may improve response validity, but reduce outcome variance.

The trial will assess a range of potentially important outcomes; however, it remains speculative as to what the most appropriate outcomes are and what effect sizes one may expect. As such, the trial remains exploratory in its nature, with the prospect of informing a larger definitive trial. The findings may help not only to test the effectiveness of the volunteering scheme, but also to understand better the underlying processes of potential changes in patients. The qualitative analyses accompanying the trial will be central to this.

If the trial findings suggest that one-to-one volunteer support for patients is effective, a larger trial may be designed to test effectiveness across different contexts and settings. Positive findings would also be a further reason to organise and support volunteering schemes which, in the UK, are more often run by voluntary organisations than NHS. Such schemes may have benefits for the integration of patients and acceptance of mental disorders in the wider population.
